# Preparedness is Essential for Western Pacific Islands During the COVID-19 Pandemic

**DOI:** 10.1017/dmp.2020.102

**Published:** 2020-04-16

**Authors:** Yujie Mei, Jijia Hu

**Affiliations:** China Institute of Boundary and Ocean Studies, Wuhan University, Wuhan, Hubei, China; National Collaborative Innovation Center for Territory Sovereignty and Maritime Rights, Wuhan University, Wuhan, Hubei, China; Division of Nephrology, Renmin Hospital of Wuhan University, Wuhan, Hubei, China

**Keywords:** COVID-19, SARS-CoV-2, Western Pacific Islands

## Abstract

**Objectives:**

To clarify the pandemic status in Western Pacific countries or territories.

**Methods:**

The WHO’s daily situation reports of COVID-19 were reviewed from January 20, 2020, to March 24, 2020. Changes in the infections, deaths, and the case fatality rate (CFR) in Western Pacific countries or territories were counted.

**Results:**

As of March 24, a total of 17 countries or territories had reported the presence of COVID-19 in the Western Pacific Region, 96,580 people have been infected and a total of 3502 deaths. Fifty-three percent (9/17) of these countries or territories had their first case within 2 wk since the WHO’s first report, most are China’s neighbors with a large and dense population. No other country or territory in this region reported a new infection from January 30 to February 28. However, 8 (47.0%) countries or territories have reported the first cases in 3 wk since February 28, almost all are islands. Many countries maintained a small number of infections for a long time after the first report, but a rapid increase occurred later. Deaths occurred in 8 countries with a total CFR of 3.63%, and the CFR varies widely, from 0.39% (Singapore) to 7.14% (Philippines).

**Conclusions:**

The regional spread of COVID-19 urgently requires an aggressive preparedness for the Western Pacific Islands.

The coronavirus disease 2019 (COVID-19) pandemic that first emerged in Wuhan has quickly spread to many countries.^1^ Sequencing analysis of throat swabs samples indicated a novel coronavirus, which was named SARS-CoV-2 (formerly known as 2019-nCoV).^2^ As of March 24, 2020, a total of 372,757 people have been infected and a total of 16,231 deaths globally.^1^ As SARS-CoV-2 demonstrates a clear human-to-human transmission characteristic and poses a severe threat to global health, the world health organization (WHO) declared the COVID-19 a pandemic of COVID-19 on March 11, 2020.^3^ As the first identified region, nearly 100,000 people have been diagnosed with COVID-19, and more than 3500 have died in Western Pacific countries or territories.^1^ To prevent the spread of the virus, some east Asian countries with large numbers of infections have taken unprecedented measures. Therefore, most of the recently infected cases have been reported in Europe and the Americas, the outbreak seems to have subsided in the Western Pacific Region. However, some island countries or territories have recently reported new imported and local transmission COVID-19 cases, including in Fiji, Papua New Guinea, Guam, French Polynesia, and New Caledonia.^1^ Given uneven resources for emergencies and inadequate health security capacities in Western Pacific Island countries and territories,^4^ it would cause a serious crisis if COVID-19 broke out in these areas. However, there is currently few research reporting on the pandemic status in the Western Pacific Islands.

## METHODS

We analyzed all the 63 daily situation reports of COVID-19 published by the WHO from January 20, 2020, to March 24, 2020, to review the pandemic situations in the Western Pacific Region (https://www.who.int). Data on the number of infected cases, deaths, and the time of first infection report were collected and analyzed. Sixty-three days were divided into 9 wk, and we ranked all the reporting countries or territories according to the time of the first confirmed infection. In addition, changes in infections, deaths, and the case fatality rate (CFR) in all these countries or territories were also counted and calculated.

## RESULTS

**FIGURE 1:**
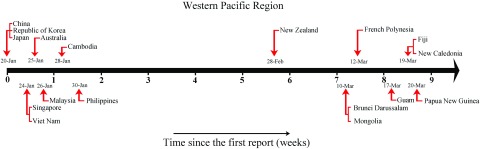
Timeline of First COVID-19 Infection Reported From Countries, Territories, or Areas in the Western Pacific Region. Match the onset time of COVID-19 infection in countries, territories, or areas in the Western Pacific Region from January 20, 2020, to March 24, 2020.

Since the WHO first reported the situation of COVID-19 on January 20, 2020, as of March 24, 2020, a total of 17 countries or territories had reported the presence of COVID-19 infection in the Western Pacific Region (Figure 1). Fifty-three percent (9/17) of them had their first case within 2 wk since January 20. Apart from Australia, these countries are mainly distributed in East Asia (China, Republic of Korea, Japan) and Southeast Asia (Singapore, Vietnam, Malaysia, Cambodia, Philippines), most are China’s neighbors with a large and relatively dense population. Surprisingly, no other countries or territories in the Western Pacific Region reported their first cases of COVID 19 for nearly a month, from January 30 to February 28. However, since February 28, a total of 8 (47.0%) nations or territories have reported the first cases within 3 wk. Notably, almost all are island countries and regions (except for Mongolia), which has brought an unprecedented crisis to the prevention and control of COVID-19 in island countries or territories.

**FIGURE 2:**
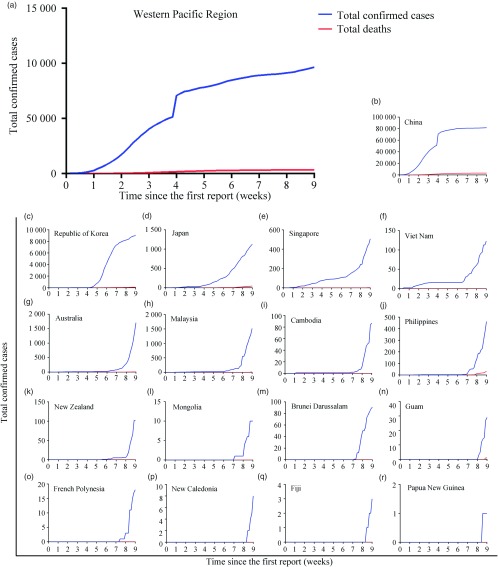
Changes in Total Infections and Deaths in Western Pacific Countries or Territories. a, Total confirmed cases and deaths in the whole Western Pacific Region. b-r, Total confirmed cases and deaths in China, Republic of Korea, Japan, Singapore, Viet Nam, Australia, Malaysia, Cambodia, Philippines, New Zealand, Brunei Darussalam, Mongolia, French Polynesia, Guam, New Caledonia, Fiji, and Papua New Guinea.

**FIGURE 3:**
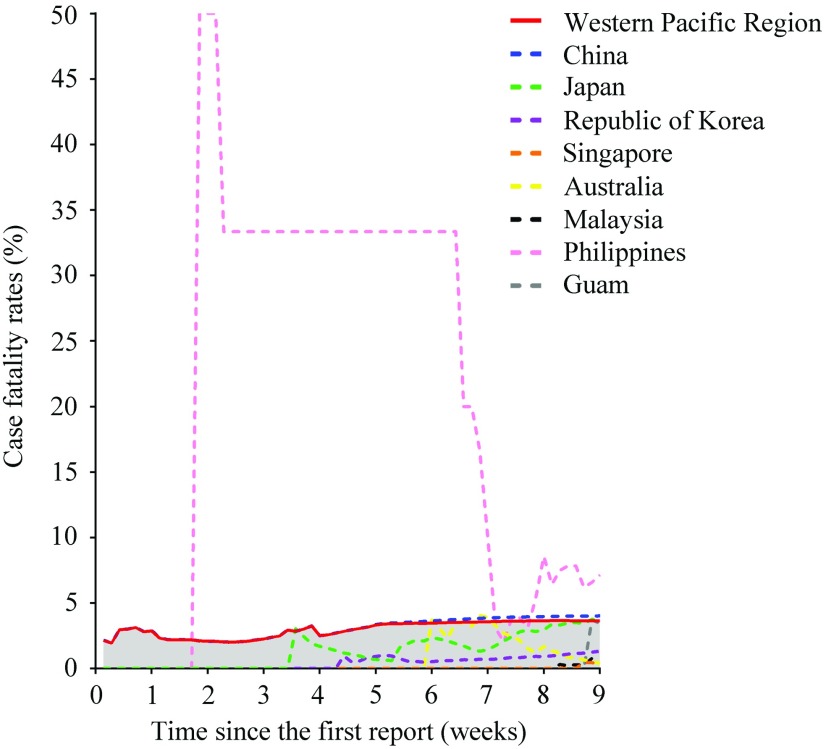
Changes in Case Fatality Rates of Western Pacific Countries or Territories.

To further clarify the transmission situation of COVID-19 in the Western Pacific Region, we analyzed the changes in the number of infections and deaths. As of March 24, 96,580 people have been infected, and a total of 3502 people have died in this region (Table 1). In addition, new infections are still on a significant rise across the region, except for China (Figure 2). Many countries maintained a small number of infections for a long time after the occurrence of the first case, but the number increased rapidly 8 wk after the WHO’s primary report (Figure 2g-j). For the CFR (Table 1; Figure 3), deaths existed in 8 countries with a total CFR of 3.63%, and the CFR varies widely, from 0.39% (Singapore) to 7.14% (Philippines).

**TABLE 1:** Data of Countries, Territories, or Areas in the Western Pacific Region With Reported Confirmed Cases of COVID-19, March 24, 2020

Country/Territory/Area	Total Confirmed Cases (*n*)	Total Deaths (*n*)	Case facility Rates (%)
China	81,747	3283	4.02%
Republic of Korea	9037	120	1.33%
Japan	1128	42	3.72%
Singapore	507	2	0.39%
Viet Nam	123	0	
Australia	1709	7	0.41%
Malaysia	1518	14	0.92%
Cambodia	87	0	
Philippines	462	33	7.14%
New Zealand	102	0	
Brunei Darussalam	91	0	
Mongolia	10	0	
French Polynesia	18	0	
Guam	29	1	3.45%
Fiji	3	0	
New Caledonia	8	0	
Papua New Guinea	1	0	
Total	96580	3502	3.63%

## DISCUSSION

We found that the pandemic situation of COVID-19 varied widely in the Western Pacific Region. Asian countries close to the epicenter of the outbreak have seen earlier cases of infection, which is consistent with the transmission pattern of SARS-CoV-2, that is, relatively frequent interpersonal interactions in the early stages of the pandemic contributed to the prevalence of COVID-19.^5,6^

Although the number of new cases seems to have leveled off across the Western Pacific Region, which may due to the timely strict measures, the suppressed infection growth rate in heavily infected countries, such as China and South Korea (Figure 2), does not indicate that transmission in these areas has been blocked. In particular, the number of reported infections is still growing in the Western Pacific islands (Figure 2). Compared with other countries in this region, there is a significant lag in the onset time of initial infected cases in these islands, possibly reflecting the remote location of and inconvenient transportation to Western Pacific Islands.

Meanwhile, there was no marked increase for a while after the first infection in many countries (Figure 2g-j), but eventually a significant increase broke out over time. This finding further proves that COVID-19 has a long incubation period and insidious initial symptoms as we described previously,^7^ which makes the disease difficult to diagnose in patients with early infection. Therefore, in cases where nucleic acid detection methods are not yet universally available, Western Pacific Islands need to be particularly vigilant for patients’ initial symptoms for COVID-19 screening.

The Western Pacific Region is home to almost 1.9 billion people across 37 countries and areas. Our previous findings suggest that the inadequate capacity to respond to public emergencies caused by an uneven distribution of medical resources is a crucial factor contributing to the unsatisfactory outcome of COVID-19 in less-developed regions.^8^ Compared with developed areas, countries or territories in the Western Pacific Region have insufficient health-care resources, and relatively low detection and prevention capacity.^4^ Although these countries or territories have just a few confirmed infected people, without adequate control and preparedness, large-scale community transmission of COVID-19 is likely to occur. Fortunately, countries such as Fiji have already started to monitor high-risk persons and strengthen the management of international borders.^9^ Given that COVID-19 does not have a high CFR if the medical system is not overloaded,^10^ it is necessary to close or strictly control the external traffic into the area and prepare adequate quarantine sites and mobile hospitals in the Western Pacific Islands to avoid a surge of patients. In addition, it is extremely important to publicize and popularize COVID-19 prevention methods, possible psychological interventions pertinent to the population exposed to the crisis, and timely disclosure of information.

The Western Pacific Islands have long been considered one of the most environmentally vulnerable regions in the world. On the one hand, the special geographical environment, such as the extreme El Nino climate, makes it difficult to establish an unbreakable health system.^11^ Furthermore, the less-developed economy and limited management capacity present additional challenges for the population to withstand an infectious disease pandemic. Economic globalization, trade, and population mobility have exacerbated the challenges of infectious disease control. Commercial and trade facilities allow infectious diseases to quickly cross the political borders of sovereign states, leading to the global spread of diseases. Due to the discontinuity between lands in the Western Pacific Island countries and territories, the extensive networks of frequent population mobility here also facilitate the spread of infectious diseases.^12,13^ Previous studies have confirmed that infectious diseases depopulated isolated Pacific islands when they were first exposed to global pathogen spread.^14^ Fortunately, a Pacific Syndromic Surveillance System (PSSS) has been established for early warning of infectious diseases.^15,16^ It is an urgent need to further improve and scientifically enable this system to inhibit the spread of COVID-19 in this special period.

In conclusion, public health crises such as COVID-19 are challenges for all mankind. The shortcomings of public health-care capacities exposed by the outbreak also highlight the urgency of strengthening control in the Western Pacific Islands.

## Availability of data and materials

All data generated or used during the study are available in the submitted article.
